# Aberrant expression and regulatory role of histone deacetylase 9 in vascular endothelial cell injury in intracranial aneurysm

**DOI:** 10.17305/bb.2023.9364

**Published:** 2024-02-01

**Authors:** Jingwei Sun, Langfeng Zhang, Quanjiang Cheng, Yajun Wu

**Affiliations:** 1 Department of Neurosurgery, Shenzhen Longhua District Central Hospital, Shenzhen, China; 2 Interventional Treatment Department, Shenzhen Longhua District Central Hospital, Shenzhen, China

**Keywords:** Intracranial aneurysm (IA), histone deacetylase 9 (HDAC9), vascular endothelial cells (VECs), miR-34a-5p, vascular endothelial growth factor-A (VEGFA), proliferation

## Abstract

Intracranial aneurysm (IA) is one of the most challenging cerebrovascular lesions for clinicians. The aim of this study was to investigate the abnormal expression and role of histone deacetylase 9 (*HDAC9*) in IA-associated injury of vascular endothelial cells (VECs). First, IA tissue and normal arterial tissue were collected and VECs were isolated from IA patients. The expression levels of HDAC9, microRNA (miR)-34a-5p, and vascular endothelial growth factor-A (VEGFA) were determined. Cell viability, proliferation, apoptosis, and migration were assessed by Cell Counting Kit-8 (CCK-8) assay, EdU staining, TUNEL staining, and transwell assay. The binding of miR-34a-5p to *VEGFA* was analyzed by the dual-luciferase assay, and the accumulation of HDAC9 and lysine histone acetylation at H3 (H3K9, H3K14, and H3K18) on the miR-34a-5p promoter was detected by the chromatin immunoprecipitation assay. The results showed that HDAC9 and VEGFA were increased and miR-34a-5p was decreased in IA tissues and cells. Silencing of HDAC9-inhibited apoptosis and increased viability, proliferation, and migration of VECs, whereas overexpression of HDAC9 exerted the opposite functions. HDAC9 accumulated at the miR-34a-5p promoter to decrease miR-34a-5p expression by reducing H3 locus-specific acetylation and further promoted VEGFA expression. Knockdown of miR-34a-5p or VEGFA overexpression reversed the protective role of HDAC9 silencing in VECs injury. In conclusion, our study suggests that HDAC9 may be a therapeutic target for IA.

## Introduction

Intracranial aneurysm (IA), a type of cerebrovascular disorder with a prevalence of approximately 3.2% in the general population, is characterized by abnormal dilation of the cerebral artery [1]. When ruptured, IA leads to subarachnoid hemorrhage, resulting in severe brain injury and even death [2]. The pathogenesis of IA has been attributed to genetic factors, hemodynamic insult, inflammatory responses, and endothelial dysfunction [3]. Endothelial cells, which are characteristic of vasoprotective, anti-inflammatory, antiatherogenic, and vasodilator functions, are very sensitive to damage caused by hemodynamic insult, nitric oxide synthase, estrogen dysregulation, and oxidative stress [4]. Vascular endothelial injury is considered the first event in the formation of IA. It leads to disruption of the internal elastic lamina, vascular thinning, and increased risk of rupture through phenotypic regulation of vascular smooth muscle cells (VSMCs) [5]. To date, microsurgical clipping and endovascular coiling are the main techniques to prevent future rupture [6], however, poor postoperative recovery cannot be ignored. Therefore, the underlying mechanism of endothelial damage should be explored to find starting points for nonsurgical treatment of IA.

Histone acetyltransferases (HATs) and histone deacetylases (HDACs) act as mediators of acetylation and deacetylation, leading to gene activation and repression, respectively [7]. There is increasing evidence that HDACs play a regulatory role in cerebrovascular diseases (e.g., stroke and ischemia-induced brain injury) as well as endothelial function [8, 9]. Histone deacetylase 9 (HDAC9), a member of class IIa of HDACs, exerts inhibitory activity in site-specific lysine (K)-histone acetylation at H3, although it does not possess strong intrinsic deacetylase activity [10]. It is noteworthy that HDAC9 is increased in IA tissues and lymphoblasts from IA patients and that silencing HDAC9 can reduce the release of proinflammatory cytokines and apoptosis in IA vascular tissues [11, 12]. Moreover, HDAC9 has been shown to induce inflammatory responses, apoptosis, and abnormal permeability of endothelial cells in cerebral ischemia/reperfusion injury [13]. However, the mechanism of HDAC9 in IA-induced endothelial injury needs further elucidation.

MicroRNAs (miRs) are conserved small non-coding RNA molecules (about 22 nucleotides in length) and interact with the 3’ untranslated regions (UTRs) of target messenger RNAs (mRNAs) to repress their expression [14]. They are crucial therapeutic targets for IA and execute the regulation of inflammation and vascular wall remodeling [15]. miR-34a has been documented as a miR signature for tumorigenesis, neuropathology, and immunomodulation [16–18]. Interestingly, miR-34a has been shown to be decreased in IA and regulate IA progression through phenotypic modulation of VSMCs [19]. Previous studies have reported that the depletion of HDAC1 leads to the upregulation of miR-34a [20, 21]. However, it is not clear whether the interaction between HDACs and miR-34a involves deacetylation at the H3 locus.

Vascular endothelial growth factor-A (VEGFA) is known for its activities in proangiogenesis, endothelial survival, and maintenance of homeostasis in developing organs [22]. VEGFA has been shown to be upregulated in ruptured IA [23] and to play a role in endothelial injury associated with various brain pathologies [24, 25]. Moreover, several databases used in this study showed the targeted binding of miR-34a-5p and *VEGFA*, indicating the involvement of miR-34a-5p/VEGFA axis in IA-induced endothelial injury. On the basis of these findings, the present study was conducted to validate the role of HDAC9 in IA-induced endothelial injury by regulating the miR-34a-5p/VEGFA axis, thereby providing a new basis for the treatment of IA.

## Materials and methods

### Collection of tissue samples

A total of 75 IA samples were microsurgically collected from 29 male and 46 female patients (age range: 31–55 years; mean age: 43.28 ± 6.39 years) treated at the Neurosurgery Department of Shenzhen Longhua District Central Hospital. Meanwhile, patients with traumatic brain injury admitted to the Neurosurgery Department were included as a control group. These were 75 cases of normal intracranial arteriole tissue collected by traumatic surgery or internal decompression (43 male and 32 female patients, age range: 34–56 years; mean age: 47.14 ± 9.68 years). Patients with a history of hypertension, diabetes, or tumors were excluded from the study. There was no difference in sex and age between the IA group and the control group (*P* > 0.05). Some of the samples were preserved in a liquid nitrogen container and were transferred to low-temperature freezer at −80 ^∘^C for subsequent testing. The remaining samples were used for the separation of vascular endothelial cells (VECs).

### Separation and culture of vascular endothelial cells (VECs)

VECs were separated and cultured from normal intracranial arteriole tissue and IA tissue. First, the tissue was cut into 3 mm^2^ pieces and incubated with 0.1% collagenase B/0.1% amylase (Roche, Basel, Switzerland) for 25 min. Then, the tissue pieces were separated, triturated with a 2 mL pipette for 2 min, and filtered with a 100 µm strainer (BD Biosciences, NJ, USA) to separate the VECs. The cell suspension was then centrifuged and resuspended in the MV2 medium containing growth factors and 20% fetal bovine serum (PromoCell, Heidelberg, Germany). Cells were then seeded in a culture dish coated with fibronectin (Sigma Aldrich Inc., St. Louis, MO, USA) at a density of 104 cells/cm^2^ (1 µg/cm^2^) and cultured with 5% CO_2_ for one day. The next day of seeding, cells were rinsed with phosphate-buffered saline (PBS) to remove nonadherent cells and placed in fresh medium. When 80%–100% confluence was achieved, immunoseparation was performed with Ulex europaeus agglutinin I (UEA; Vector Laboratories, Ltd., Peterborough, UK) coated beads (Dynabeads M-450 Tosylactivated, Oxoid, Hampshire, UK) to obtain purified VECs. VECs bound to the beads were aggregated using a magnetic particle concentrator, while unbound VECs were removed by washing twice with the base medium. UEA-positive cells were resuspended in the medium and seeded in fibronectin-coated medium to improve adherence and growth. Confluence was achieved after 4–6 days of culture.

### Characterization of VECs

Immunocytochemical staining was performed with antibodies against CD31 and von Willebrand factor (vWF). First, cells were washed twice with PBS, fixed with 4% paraformaldehyde, and incubated with 3% H_2_O_2_ for 10–15 min to quench endogenous peroxidase activity. They were then incubated with 0.1% Triton X-100 for 10 min until the cells were perforated. Cells were dripped with specific primary antibodies against vWF (ab6994, Abcam, Cambridge, MA, USA) and CD31 (ab76533, Abcam) for overnight incubation at 4 ^∘^C and then incubated with the secondary antibody against immunoglobulin G (IgG; ab205718, Abcam) for 45 min at 37 ^∘^C in the dark. Visualization was performed with 3,3’-diaminobenzidine for 4 min in the dark and was terminated with the addition of distilled water. Cells were then observed under an inverted phase contrast microscope.

### Treatment of VECs

VECs were divided into the control group (nontransfected VECs derived from normal tissue) and the IA group (nontransfected VECs derived from IA tissue). To investigate the effects of HDAC9 (Homo sapiens, transcript variant 6; GeneID: 9734), miR-34a-5p (Homo sapiens; GeneID: 407040), and VEGFA (Homo sapiens, transcript variant 1; GeneID: 7422) on the VECs, the cells of the IA group were transfected. *HDAC9* and *VEGFA* sequences were inserted into pcDNA3.1 vectors (Invitrogen, Carlsbad, CA, USA) to generate overexpressed *HDAC9* and *VEGFA* vectors (oe-HDAC9 and oe-VEGFA). miR-34a-5p inhibitor and negative control inhibitor were synthesized by GenePharma Crop (Shanghai, China). Lipofectamine 3000 (Invitrogen) was used to transfect the cells. In brief, group IA VECs were cultured to 80% confluence, and then the appropriate transfection material and Lipofectamine 3000 were added. After 48 h, the transfected cells were collected.

### Cell Counting Kit-8 (CCK-8) assay

Transfected VECs were separated with trypsin and prepared into a cell suspension (2 × 104 cells/mL) and seeded into the 96-well plate at the concentration of 100 µL/well. After 48 h of culture at 37^∘^C, 10 µL CCK-8 solution (Beyotime, Jiangsu, China) was added to each well, followed by another 2 h of culture. Finally, the proliferation rate of VECs was calculated based on the optical density value at 450 nm, which was determined using a microplate reader (Promega, Madison, WI, USA).

### EdU staining

Cell proliferation was evaluated using the Click-iT EdU imaging assay kit (Thermo Fisher Scientific, Carlsbad, CA, USA). VECs were incubated with 100 µM EdU solution for 2 h, fixed with 4% paraformaldehyde for 20 min, incubated with 2% glycine for 15 min, washed twice with PBS, and penetrated with 150 µL penetrant through a fluorescence microscope (Olympus, Tokyo, Japan) to observe five random fields of view. All cells were visualized with blue fluorescence, and EdU-permeated replicating cells were visualized with red fluorescence. The proliferation rate was calculated as the number of blue fluorescent cells/red fluorescent cells × 100%.

### TUNEL assay

According to the provided instructions, the state of apoptosis was assessed using the one-step TUNEL apoptosis assay kit (Beyotime). VECs were imaged by fluorescence microscopy and counted under five random fields of view. Cell apoptosis was assessed by the percentage of TUNEL-positive cells to total cells.

### Transwell assays

The 24-well plates were placed in 6.5 mm transwell chambers (pore diameter 8 µm). VECs were trypsinized and serum-free medium was added. Then, the apical chamber was filled with cell suspension at a ratio of 100–300 µL/chamber with a number of 104 cells. After 24 h of culture, cells in the apical chamber were discarded, and migrated cells were fixed with methanol:glacial acetic acid (3:1) at a concentration of 500 µL/well, followed by 30–60 min of staining with Giemsa staining solution (mixing ratio 1:9). The number of cells under five random fields of view was counted under an inverted microscope.

### Chromatin immunoprecipitation (ChIP) assay

The ChIP assay was performed according to the protocol provided (Millipore, Bedford, MA, USA). VECs were trypsinized and fixed with 1% formaldehyde to cross-link DNA and protein. Then, the compound was shredded with ultrasound to generate fragments and centrifuged at 13,000 g and 4 ^∘^C. The collected supernatant was incubated with IgG antibodies (ab6757, Abcam), HDAC9 (ab59718, Abcam), H3 lysine 9 acetylation (H3K9ac; ab32129, Abcam), H3 lysine 14 acetylation (H3K14ac; ab52946, Abcam), and H3 lysine 18 acetylation (H3K18ac; ab40888, Abcam) overnight at 4 ^∘^C. Then, the endogenous DNA-protein compound was precipitated with agarose/sepharose protein. The supernatant was aspirated and discarded after a short centrifugation, after which the non-specific compounds were washed at 65 ^∘^C to de-link overnight. DNA fragments were recovered by phenol/chloroform-dependent extraction and purification. Expression of the miR-34a promoter (relative to IgG) was determined by real-time quantitative polymerase chain reaction (RT-qPCR). Information on PCR primers is provided in Table 1.

**Table 1:** Sequence information of PCR primers

**Gene**	**Sequence (5’-3’)**
*HDAC9*	F: TTCTGTGGCTGCTGTAGGAT
	R: TAAAGGTGAGATGGGCTCCAG
miR-34a-5p	F: GAAGCGCTGGCAGTGTCTTAG
	R: ACTGGTGTCGTGGAGTCGGCA
*VEGFA*	F: CGAAAGCGCAAGAAATCCCG
	R: CTCCAGGGCATTAGACAGCA
*U6*	F: GCTCGCTTCGGCAGCACATATA
	R: GGAACGCTTCACGAATTTGCG
*GAPDH*	F: GGTCCCAGCTTAGGTTCATCA
	R: AATCCGTTCACACCGACCTT
miR-34a promoter	F: CCTGTCAACTCCAACGGGAC
	R: GTCAGCAGGACACAGGACAC

*HDAC9*: Histone deacetylase 9; miR: microRNA; *VEGFA*: Vascular endothelial growth factor-A; *U*6: U6 small nuclear RNA; *GAPDH*: Glyceraldehyde-3-phosphate dehydrogenase.

### Dual-luciferase assay

The downstream target genes of miR-34a-5p were predicted using the Starbase database (http://starbase.sysu.edu.cn/index.php) [26], the RNA22 database (https://cm.jefferson.edu/rna22/Interactive/) [27], the miRTarBase database (https://mirtarbase.cuhk.edu.cn/∼miRTarBase/miRTarBase_2019/php/index.php) [28], and the miRWalk database (http://mirwalk.umm.uni-heidelberg.de/) [29]. According to the target site of miR-34a-5p and *VEGFA*, the 3’-UTR sequence of *VEGFA* was amplified and inserted into the pmiR-RB-report vector (OBIO, Shanghai, China) to generate a wild-type (WT) luciferase vector, and the mutant sequence was used to construct a mutant type (MUT) luciferase vector. VECs (*n* ═ 1.2 × 104) in the IA group in 96-well plate were co-transected with miR-34a-5p mimic or mimic NC (RiboBio, Guangdong, China) and WT or MUT vector. After 48 h of transfection, luciferase activity was quantified using the dual-luciferase reporter gene analysis system (Promega). Firefly luciferase activity was normalized with Renilla luciferase activity.

### RT-qPCR

Total RNA was separated from tissues and cells using TRIzol reagent (Invitrogen, China). Using the Revert Aid first-strand complementary DNA (cDNA) kit (Thermo Fisher Scientific, Waltham, MA, USA), RT-PCR was used to generate cDNA. Determination of qPCR was performed with Power Track SYBR Green Master Mix, using glyceraldehyde-3-phosphate dehydrogenase (*GAPHD*) or *U6* [30] for normalization. The relative expression levels were calculated using the 2^−ΔΔCt^ method [31]. The primers used are listed in Table 1.

### Western blot assay

Cells and tissues were treated with radioimmunoprecipitation assay lysis buffer (Abcam) to extract total protein and treated with bicinchoninic acid (Beyotime) to quantify protein concentration. After 10% sodium dodecyl sulfate–polyacrylamide gel electrophoresis-dependent separation, protein samples were transferred to polyvinylidene fluoride membranes (Bio-Rad, China) and blocked with 5% bovine serum albumin. After these steps, the membranes were treated with primary antibodies diluted with blocking buffer in a shaker at 4 ^∘^C overnight. The next day, the primary antibodies were washed away with Tris-Buffered Saline Tween (TBST), followed by incubation of the membranes with specific secondary antibodies in a shaker for 2 h. After another wash with TBST, signaling was detected with the enhanced chemiluminescence kit, using NIH Image J software (version 1.52a; National Institutes of Health, Bethesda, MD, USA) to analyze gray-scale values. The antibodies used in this assay were as follows: HDAC9 (1:10000, ab109446, Abcam), VEGFA (1:450, ab183100, Abcam), GAPDH (1:2500, ab9485, Abcam), and secondary antibody (1:2000, ab205718, Abcam).

### Ethical statement

This study was ratified by the Review Committee of Shenzhen Longhua District Central Hospital (approval number 2019-006-01) and followed the Declaration of Helsinki. All participants signed an informed consent form.

### Statistical analysis

SPSS21.0 software (IBM SPSS Statistics, Chicago, IL, USA) and GraphPad Prism 8.0 software (GraphPad Software Inc., San Diego, CA, USA) were used for statistical analysis and plotting. The relevant tests showed that the data conformed to the normal distribution and homogeneity of variance. For the analysis of statistical significance between two panels, the *t*-test was used, and for the analysis of statistical significance between multiple panels, one-way or two-way analysis of variance (ANOVA) was used, followed by Tukey’s multiple comparison test. *P* values were determined by a two-sided test. *P* < 0.05 was indicative of statistical significance, and *P* < 0.01 was indicative of extreme statistical significance.

## Results

### HDAC9 expression is increased in IA tissues and cells

**Figure 1.**
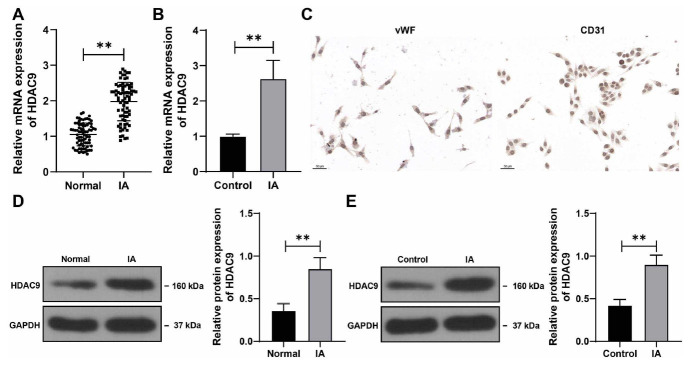
**HDAC9 expression is increased in IA tissues and cells.** **P < 0.01; *n* ═ 75. Cell experiments were performed three times independently. Data in figures (B), (D), and (E) are presented as mean ± standard deviation. Pairwise comparisons were analyzed by the *t*-test. (A and B) mRNA levels of *HDAC9* in IA tissue/VECs and normal tissue/control VECs were determined by RT-qPCR; (C) VECs were separated and characterized by immunocytochemical staining for detection of vWF and CD31; (D and E) Protein levels of HDAC9 in IA tissue/VECs and normal tissue/control VECs were determined by the western blot assay. HDAC9: Histone deacetylase 9; IA: Intracranial aneurysm; VEC: Vascular endothelial cell; RT-qPCR: Real-time quantitative polymerase chain reaction; vWF: von Willebrand factor.

We examined the expression pattern of HDAC9 in the collected tissues. The result of RT-qPCR showed that the mRNA levels of *HDAC9* were significantly higher in IA tissues compared with normal tissues (*P* < 0.01, Figure 1A). The result of the western blot assay showed the same trend (*P* < 0.01, Figure 1D). We then separated the VECs from the tissues and analyzed the expression of vWF and CD31 by immunocytochemical staining. The results showed that the VECs were covered by large amounts of brown particles and the positive rates of vWF and CD31 were up to 95% (Figure 1C), confirming that the separated cells were VECs. Next, we measured the expression levels of HDAC9 in the cells and found that HDAC9 expression levels were significantly higher in the VECs in the IA group compared with the control group (*P* < 0.01, Figure 1B and 1E). These results suggest that HDAC9 expression was increased in IA tissues and cells.

### Downregulation of HDAC9 inhibits apoptosis and promotes proliferation and migration of IA VECs

**Figure 2.**
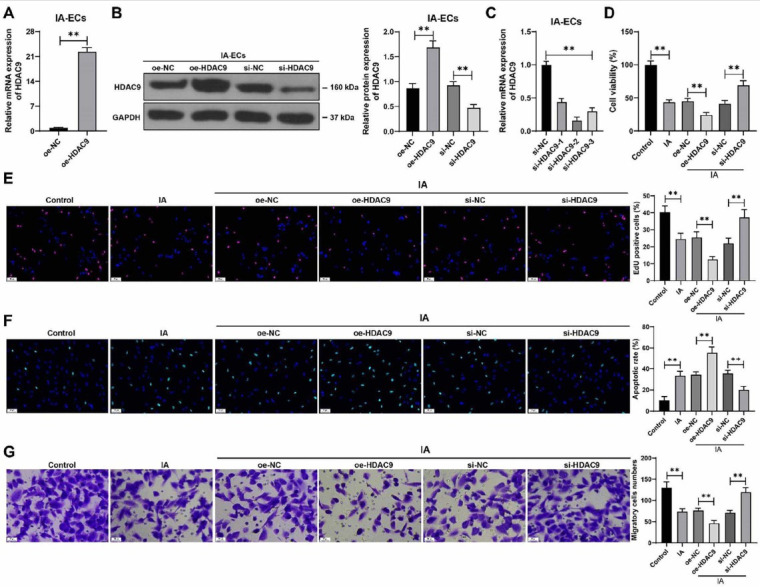
**HDAC9 downregulation inhibits the apoptosis and promotes the proliferation and migration of IA VECs.** **P < 0.01. Cell experiments were performed three times independently. Data are presented as mean ± standard deviation. Pairwise comparisons in (A) were analyzed by the *t*-test. Multiple comparisons in (B–G) were analyzed by one-way ANOVA, followed by Tukey’s multiple comparison test. VECs in the IA group were transfected with oe-HDAC9, with the cells transfected with oe-NC as the negative control; VECs in the IA group were transfected with si-HDAC9, with the cells transfected with si-NC as the negative control. (A–C) HDAC9 expression levels in IA VECs were determined by RT-qPCR and western blot assay; (D) Cell viability was evaluated by CCK-8 assay; (E) Cell proliferation was assessed by EdU staining (see Figure S1 for detailed images); (F) Cell apoptosis was assessed by TUNEL staining (see Figure S2 for detailed images); (G) Cell migration was tested by transwell assay. HDAC9: Histone deacetylase 9; IA: Intracranial aneurysm; VEC: Vascular endothelial cell; ANOVA: Analysis of variance; CCK-8: Cell Counting Kit-8; RT-qPCR: Real-time quantitative polymerase chain reaction; EDU: 5-ethynyl-2'-deoxyuridine; TUNEL: TdT-mediated dUTP nick end labeling.

Next, the expression of HDAC9 was upregulated in IA VECs by transfection with oe-HDAC9 (*P* < 0.01, Figure 2A and 2B). Moreover, the expression of HDAC9 in IA VECs was downregulated by transfection with si-HDAC9 (*P* < 0.01, Figure 2B and 2C), and si-HDAC9-2 with better knockdown efficiency was applied to the subsequent assays. After overexpression of HDAC9, the cell proliferation potential was weakened and the number of EdU-positive cells was reduced, which was in contrast to the results caused by the downregulation of HDAC9 (*P* < 0.01, Figure 2D and 2E). Moreover, overexpression of HDAC9 promoted apoptosis of IA VECs, and downregulation of HDAC9 suppressed apoptosis (*P* < 0.01, Figure 2F). Similarly, oe-HDAC9 treatment inhibited the migration of IA VECs, whereas si-HDAC9 exerted the opposite effect (*P* < 0.01, Figure 2G). Our results suggest that the downregulation of HDAC9 inhibits apoptosis and promotes proliferation and migration of IA VECs.

### HDAC9 is enriched at the miR-34a-5p promoter and inhibits miR-34a-5p expression

**Figure 3.**
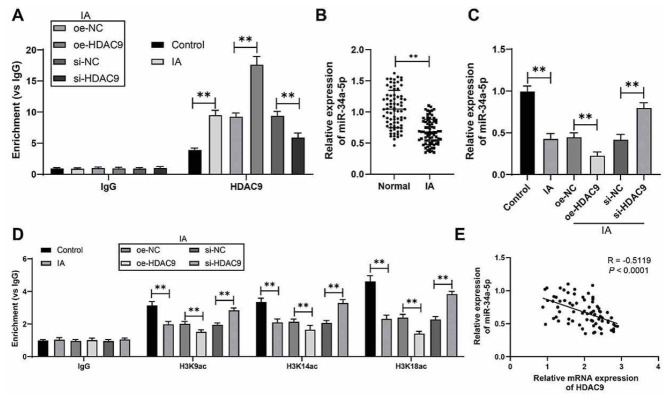
**HDAC9 is enriched in the miR-34a-5p promoter and inhibits miR-34a-5p expression.** **P < 0.01. Data in (A), (C), and (D) are presented as mean ± standard deviation. Pairwise comparisons in (B) were analyzed by the *t*-test; multiple comparisons in (C) were analyzed by one-way ANOVA and in (A) and (D) were analyzed by two-way ANOVA, followed by Tukey’s multiple comparison test. (A) The enrichment of HDAC9 on the miR-34a-5p promoter (vs IgG) was analyzed by the ChIP assay; (B and C) miR-34a-5p levels in tissues (*n* ═ 75) and cells were determined by RT-qPCR; (D) The enrichment of H3K9ac, H3K14ac, and H3K18ac on the miR-34a-5p promoter (vs IgG) was analyzed by the ChIP assay; (E) Correlation between HDAC9 and miR-34a-5p was analyzed by Pearson correlation analysis. Cell experiments were performed three times independently. HDAC9: Histone deacetylase 9; miR**:** microRNA; ANOVA: Analysis of variance; ChIP: Chromatin immunoprecipitation; RT-qPCR: Real-time quantitative polymerase chain reaction; H3K9ac: H3 lysine 9 acetylation; H3K14ac: H3 lysine 14 acetylation; H3K18ac: H3 lysine 18 acetylation; IgG: Immunoglobulin G.

There is evidence that HDAC9 is enriched at the miR-20a promoter and inhibits miR-20a expression [32]. It has been reported that miR-34a is regulated by HDAC1 and downregulated at IA [19, 20]. In view of this, we speculated that miR-34a-5p is involved in the downstream mechanism of HDAC9. The result of ChIP assay showed that HADC9 can be enriched at the miR-34a promoter and that this enrichment was increased by overexpression of HADC9 and decreased by downregulation of HADC9 (*P* < 0.01, Figure 3A). HDAC9 can modify H3 locus (H3K9, H3K14, and H3K18)-specific lysine histone acetylation [10]. It was observed that the accumulation of H3K9ac, H3K14ac, and H3K18ac was reduced by overexpression of HDAC9, which was reversed by downregulation of HDAC9 (*P* < 0.01, Figure 3D). We determined the expression of miR-34a-5p, and the results showed that the expression levels of miR-34a-5p were significantly lower in IA tissues and VECs compared with normal tissues and VECs (*P* < 0.01, Figure 3B and 3C) and had a negative correlation with *HDAC9* mRNA levels in IA tissues (*P* < 0.01, Figure 3E). Taken together, these results indicated that HDAC9 was enriched at the miR-34a-5p promoter and inhibited miR-34a-5p expression by reducing H3 locus-specific acetylation modification.

### miR-34a-5p downregulation moderates the protective role of HDAC9 downregulation in IA-associated VECs injury

**Figure 4.**
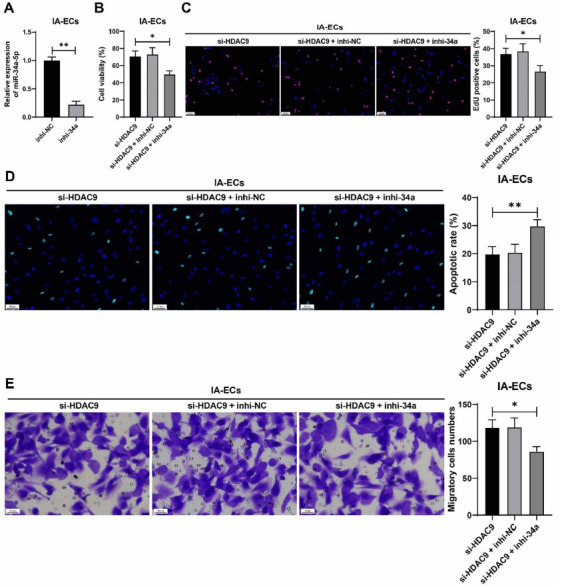
**miR-34a-5p downregulation moderates the protective role of HDAC9 downregulation in IA-associated VECs injury.** *P < 0.05, **P < 0.01. VECs in the IA group were transfected with miR-34a-5p inhibitor (inhi-34a), with the cells transfected with inhi-NC as the negative control. Cell experiments were performed three times independently. Data are presented as mean ± standard deviation. Pairwise comparisons in (A) were analyzed by the *t*-test; multiple comparisons in (B–E) were analyzed by one-way ANOVA, followed by Tukey’s multiple comparison test. (A) miR-34a-5p levels in IA VECs were determined by RT-qPCR; (B) Cell viability was assessed by CCK-8 assay; (C) Cell proliferation was assessed by EdU staining (see Figure S3 for detailed images); (D) Cell apoptosis was assessed by TUNEL staining (see Figure S4 for detailed images); (E) Cell migration was tested by transwell assay. miR: microRNA; HDAC9: Histone deacetylase 9; IA: Intracranial aneurysm; VEC: Vascular endothelial cell; ANOVA: Analysis of variance; RT-qPCR: Real-time quantitative polymerase chain reaction; CCK-8: Cell Counting Kit-8; EDU: 5-ethynyl-2'-deoxyuridine; TUNEL: TdT-mediated dUTP nick end labeling.

To examine the effect of miR-34a-5p on IA-associated VECs injury, miR-34a-5p expression was downregulated in IA VECs by transfection with a miR-34a-5p inhibitor (inhi-34a) (*P* < 0.01, Figure 4A), and the transfected cells were treated with si-HDAC9. Our results showed that after the downregulation of miR-34a-5p, cell viability and the number of EdU-positive cells were reduced (*P* < 0.05, Figure 4B and 4C). Moreover, knockdown of miR-34a-5p promoted apoptosis of IA VECs (*P* < 0.01, Figure 4D). Compared with the si-HDAC9 group, the migration of IA VECs was suppressed in the si-HDAC9 + inhi-34a group (*P* < 0.05, Figure 4E). In general, these results suggest that miR-34a-5p downregulation may attenuate the protective role of HDAC9 downregulation in IA-associated VECs injury.

### miR-34a-5p targets and inhibits VEGFA expression

**Figure 5.**
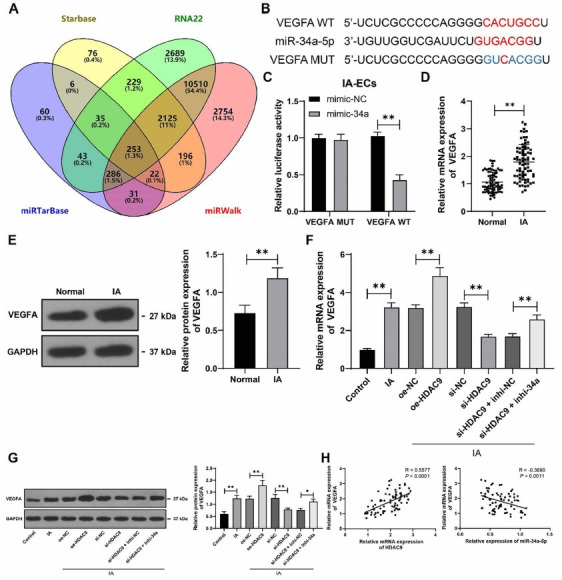
**miR-34a-5p targets and inhibits VEGFA expression.** *P < 0.05, **P < 0.01. Cell experiments were performed three times independently. Data in (B) and (E–G) are presented as mean ± standard deviation. Pairwise comparisons in (D) and (E) were analyzed by the *t*-test; multiple comparisons in (F) and (G) were analyzed by one-way ANOVA and in (C) were analyzed by two-way ANOVA, followed by Tukey’s multiple comparison test. (A) The downstream target genes of miR-34a-5p were predicted by multiple databases and their intersections were identified; (B and C) Binding site and mutant site of miR-34a-5p and *VEGFA* and their targeted binding were validated by the dual-luciferase assay; (D–G) VEGFA expression levels in tissues (*n* ═ 75) and cells were determined by RT-qPCR and western blot assay; (H) Correlation between *HDAC9*/miR-34a-5p and *VEGFA* was analyzed by Pearson correlation analysis. miR: microRNA; VEGFA: Vascular endothelial growth factor-A; ANOVA: Analysis of variance; RT-qPCR: Real-time quantitative polymerase chain reaction; HDAC9: Histone deacetylase 9.

To further investigate the downstream mechanism of miR-34a-5p, the downstream target genes of miR-34a-5p were predicted from multiple databases, and the intersections in the Venn diagram were identified (Figure 5). Among the genes in the crossing points, we paid attention to VEGFA, whose increase in IA has been previously documented [23]. Therefore, we selected *VEGFA* as the downstream target gene of miR-34a-5p. Dual-luciferase assay confirmed the targeted binding between miR-34a-5p and *VEGFA* (*P* < 0.01, Figure 5B and 5C). VEGFA was found to be significantly increased in IA tissues and VECs (*P* < 0.01, Figure 5D–5G). Moreover, *VEGFA* expression was significantly increased by overexpression of HDAC9 and inhibited by downregulation of *HDAC9*, whereas miR-34a-5p silencing counteracted the inhibition of VEGFA expression (*P* < 0.05, Figure 5F and 5G). In IA tissues, *VEGFA* mRNA was positively correlated with *HDAC9* mRNA but negatively correlated with miR-34a-5p level (*P* < 0.01, Figure 5H). The above data illustrate the miR-34a-5p-driven inhibition of VEGFA.

### Overexpression of VEGFA counteracts the protective role of HDAC9 downregulation in IA-associated VECs injury

**Figure 6.**
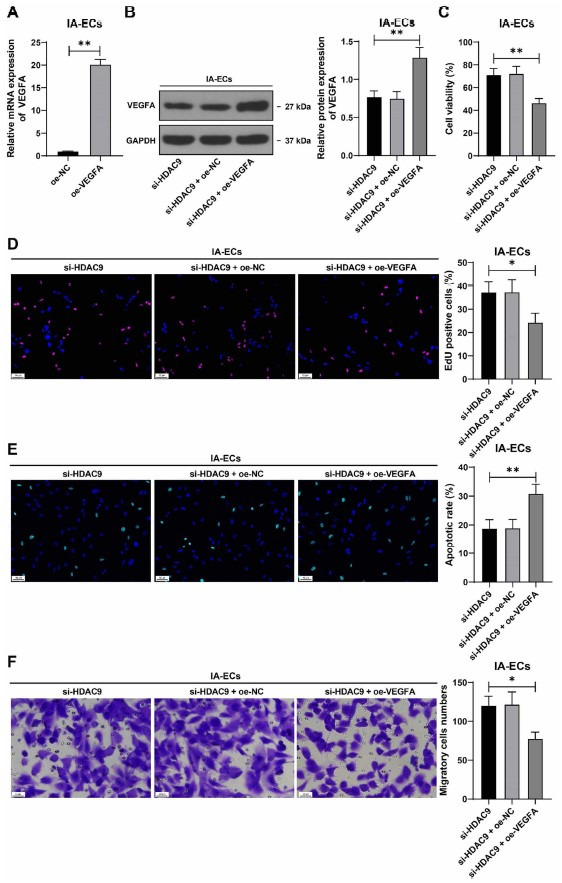
**VEGFA overexpression counteracts the protective role of HDAC9 downregulation in IA-associated VECs injury.** *P < 0.05, **P < 0.01. VECs in the IA group were transfected with oe-VEGFA, with the cells transfected with oe-NC as the negative control. Cell experiments were performed three times independently. Data are presented as mean ± standard deviation. Pairwise comparisons in (A) were analyzed by the *t*-test; multiple comparisons in (B–F) were analyzed by one-way ANOVA, followed by Tukey’s multiple comparison test. (A and B) VEGFA expression levels in IA VECs were determined by RT-qPCR and western blot assay; (C) Cell viability was assessed by CCK-8 assay; (D) Cell proliferation was assessed by EdU staining (see Figure S5 for detailed images); (E) Cell apoptosis was assessed by TUNEL staining (see Figure S6 for detailed images); (F) Cell migration was tested by transwell assay. VEGFA: Vascular endothelial growth factor-A; HDAC9: Histone deacetylase 9; IA: Intracranial aneurysm; VEC: Vascular endothelial cell; RT-qPCR: Real-time quantitative polymerase chain reaction; CCK-8: Cell Counting Kit-8; EDU: 5-ethynyl-2'-deoxyuridine; TUNEL: TdT-mediated dUTP nick end labeling.

Finally, to examine the effect of VEGFA on IA-associated VECs injury, the expression of VEGFA in cells was increased by transfection with oe-VEGFA (*P* < 0.01, Figure 6A and 6B), and the transfected cells were treated with si-HDAC9. After overexpression treatment of VEGFA, cell proliferation potential was attenuated and the number of EdU-positive cells was reduced (*P* < 0.05, Figure 6C and 6D), cell apoptosis was enhanced (*P* < 0.01, Figure 6E), and migration of IA VECs was inhibited (*P* < 0.05, Figure 6F). Our results suggest that overexpression of VEGFA may counteract the protective role of HDAC9 downregulation in IA-associated VECs injury.

## Discussion

**Figure 7.**
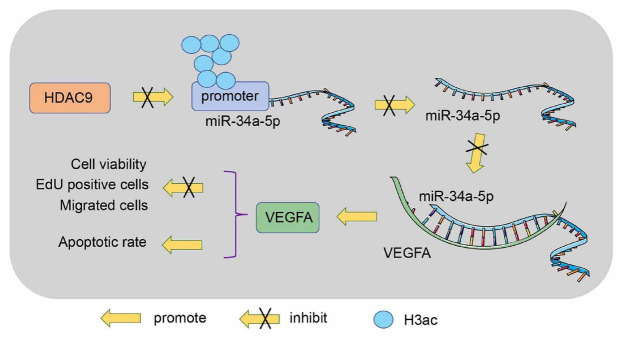
**Regulatory role of HDAC9 in VECs injury in IA**. HDAC9 was upregulated in IA and was enriched on the miR-34a-5p promoter and repressed miR-34a-5p expression by reducing lysine histone acetylation at H3 locus (H3K9, H3K14, and H3K18), further promoting VEGFA expression and exacerbating VECs injury in IA. HDAC9: Histone deacetylase 9; VEC: Vascular endothelial cell; IA: Intracranial aneurysm; miR**:** microRNA; H3K9: H3 lysine 9 acetylation; H3K14: H3 lysine 14 acetylation; H3K18: H3 lysine 18 acetylation; VEGFA: Vascular endothelial growth factor-A.

IA is a cerebrovascular disorder and leads to subarachnoid hemorrhage when ruptured [3]. Endothelial injury is a key pathologic event of IA and leads to an increased risk of rupture [4]. Data are accumulating that the targeted use of HDACs offers a bright prospect for the treatment of cerebrovascular disease [9]. Moreover, the interplay between HDACs and their target miRs plays a fundamental role in epigenetic regulation of diseases [33]. By exploring the novel HDACs-miRs axis in IA, our study provided strong evidence that HDAC9 represses miR-34a-5p by reducing lysine histone acetylation at H3 (H3K9, H3K14, and H3K18) and further promotes VEGFA expression, thereby exacerbating endothelial injury in IA (Figure 7).

Overexpression of HDAC9 has been demonstrated in clinical samples from IA and in the rat model [11], which is consistent with its expression pattern in ischemic brain injury [32]. Similarly, silencing HDAC9 exerts a neuroprotective effect in brain injury by suppressing neuronal inflammation and apoptosis [32, 34]. This suggests that HDAC9 expression is not only an indicator of aneurysm formation but also related to brain injury in the rupture of IA. Moreover, overexpression of HDAC9 has been shown to induce oxidized low-density lipoprotein or oxygen-glucose deprivation-induced injury of cerebral VECs [13, 35], indicating the detrimental role of HDAC9 in endothelial cell survival in IA cases, especially in cases related to vascular risks, such as atherosclerosis and cerebral ischemia. Our results suggest that HDAC9 was upregulated in IA tissues from patients and IA tissue-derived VECs and that silencing of HDAC9 inhibited apoptosis and promoted proliferation and migration of VECs. Nevertheless, the role of HDACs in vascular endothelial injury is not always consistent, and activation of some HDACs (e.g., HDAC4) may attenuate VEC injury [36]. Because currently available HDAC inhibitors are pan-inhibitors, specific inhibition of HDAC9 in IA should be achieved by exploring the upstream mechanism rather than using HDAC inhibitors.

The interaction between HDACs and miRs plays a regulatory role in numerous human diseases. Among HDACs, HDAC9 is specialized in H3 locus-specific acetylation. It was found that the upregulation of miR-34a is triggered by the silencing of HDAC1 [20]. In contrast, overexpression of miR-34a was shown to reduce HDAC1-mediated H3K9ac [37]. Our data suggest that HDAC9 is enriched at the miR-34a promoter and can reduce the enrichment of H3K9ac, H3K14ac, and H3K18ac at the promoter, resulting in decreased miR-34a-5p expression. A pioneering study has documented the involvement of miR-34a in the phenotypic regulation of VSMCs to influence progression IA [19]. Since VSMCs can induce the functional change of VECs [38], miR-34a could also regulate the functions of VECs. Our subsequent results showed that inhibition of miR-34a-5p induced the injury of VECs. However, Supriya et al. [39] reported that miR-34a-5p was upregulated in the plasma of patients with ruptured IA, and Liao et al. [40] claimed that suppression of miR-34a had a protective effect on the survival of ischemia-induced VECs. These conflicting evidences may be caused by different tissue samples and stimulations of VECs. Overall, our results suggest that HDAC9 plays a detrimental role in the injury of VECs by suppressing miR-34a-5p through histone acetylation at H3, but it is important to provide more clarity on the expression and role of miR-34a-5p.

In addition, we explored the downstream mechanism of miR-34a-5p and narrowed it down onto VEGFA. VEGFA has been shown to be one of the upregulated angiogenic factors in ruptured IA [41] and can accelerate injury by intracerebral hemorrhage injury [42]. However, many studies have demonstrated the protective role of VEGFA in brain disorders, such as cerebral ischemia/reperfusion injury and traumatic brain injury [43, 44]. Li et al. have revealed the binary functional pattern of VEGFA in brain injury, with the elevation exerting adverse effects in the onset and early progression and later beneficial effects later in recovery [45], which may explain its controversial role in brain injury. The interaction between miR-34a-5p and VEGFA has been documented in hepatocellular carcinoma, glioma, and endometriosis [46–48]. In this study, we tested the binding relationship between miR-34a-5p and *VEGFA* 3’-UTR using the dual-luciferase assay. *VEGFA* showed a positive correlation with *HDAC9* and an inverse correlation with miR-34a-5p, suggesting that miR-34a-5p negatively regulates VEGFA by binding to *VEGFA* 3’-UTR. Our subsequent experiments indicated that overexpression of VEGFA triggered the injury of VECs. Accordingly, sevoflurane-induced suppression of VEGFA may exert a protective function role for cerebral endothelial barrier function and structure [49]. Similarly, it is likely that VEGF antagonism attenuates apoptosis, oxidative stress, and endoplasmic reticulum stress and promotes the proliferation of VECs, thereby attenuating ischemia/reperfusion-induced brain injury [50]. Nevertheless, most previous literatures have demonstrated the protective role of VEGFA in brain endothelial injury. We are the first to reveal the detrimental role of VEGFA in IA-associated endothelial injury.

## Conclusion

Our study demonstrated for the first time that HDAC9 induces H3 deacetylation-dependent suppression of miR-34a-5p and upregulation of VEGFA, thereby promoting endothelial injury at IA, suggesting that HDAC9 may be a potential therapeutic target for IA. However, our mechanism has only been validated at the cellular level without in vivo validation and was warranted to be used for clinical trials. Moreover, the upstream mechanism of HDAC9 is still unknown. Our investigation of the downstream mechanism of miR-34a-5p is limited to VEGFA, regardless of the many downstream targets of miR-34a-5p. Since HDAC9 can form a complex with HDAC1 and inhibition of HDAC1 can lead to an increase in acetylation levels of H3K9 and H3K14 [51], the HDAC1–HDAC9 complex may play a role in IA. In the future, we will validate our mechanism by animal experiments and explore other mechanisms of HDAC9 and the role of HDAC1–HDAC9 complex to provide current theoretical insights for the treatment of IA.

## Supplemental data

Supplementary data are available at the following link: https://www.bjbms.org/ojs/index.php/bjbms/article/view/9364/2880
